# Sexual Dimorphism in Three Populations of the Chiala Mountain Salamander, *Batrachuperus karlschmidti* (Caudata: Hynobiidae)

**DOI:** 10.3390/ani16020332

**Published:** 2026-01-21

**Authors:** Xiuying Liu, Chunhao Shen, Yuanhua Xu, Jian Song, Min Lou, Jianli Xiong

**Affiliations:** 1 School of Geography and Environment, Mianyang Normal University, Mianyang 621000, China; csfulxy@126.com; 2 School of Life Sciences (School of Ecological Forestry), Mianyang Normal University, Mianyang 621000, China; hepenghe25@gmail.com (C.S.); yuan010120@163.com (Y.X.); songjian7654321@163.com (J.S.); 15194617178@163.com (M.L.); 3 Forest Ecology and Conservation in the Upper Reaches of the Yangtze River Key Laboratory of Sichuan Province, Mianyang Normal University, Mianyang 621000, China; 4 Key Laboratory of Research and Conservation of Biological Diversity in Minshan Mountain of National Park of Giant Pandas at Mianyang, Normal University of Sichuan Province, Mianyang 621000, China

**Keywords:** fecundity selection, mountain salamanders, sexual body dimorphism, sexual shape dimorphism, sexual selection, variation

## Abstract

Sexual dimorphism (SD) is a widespread phenomenon among animals and has attracted considerable interest in evolutionary biology. Most studies on SD have been limited to a single population, and few have focused on multiple populations. In this study, size and shape SD were evaluated in three populations of *Batrachuperus karlschmidti*, a hynobiid species endemic to China. SD was not found in body size, but was observed in body shape. Males had larger relative dimensions in head length, head width, forelimb length, forelimb width, hindlimb length, hindlimb width, and tail length. Conversely, females were larger in the relative dimension of interlimb distance. Sexual selection can account for SD in head and limbs, thereby enhancing male reproductive success. Conversely, fecundity selection drives SD in limbs, tail length, and interlimb distance, ultimately improving the reproductive ability of both sexes. Differences in sexual shape dimorphism (SShD) traits were also found among populations, which may be caused by dissimilar levels of selection forces in the environment. This study provides insight into identifying the causes that promote sexual dimorphism, as well as the degree of difference in SShD traits among populations.

## Simple Summary

In the present study, we explored the sexual dimorphism in size and shape in three populations of *Batrachuperus karlschmidti*, a hynobiid species endemic to China. The results revealed that *B. karlschmidti* exhibited obvious sexual dimorphism in body shape, but not in body size. The observed sexual shape dimorphism could be explained by the sexual selection and fecundity selection hypotheses. This study represents one of the few reported examples of sexual dimorphism among hynobiid salamander populations.

## 1. Introduction

Sexual dimorphism (SD) refers to a phenotypic difference between conspecific males and females, and is common among animals [1,2,3]. Amphibians are an ecologically and evolutionarily important group of animals that live in terrestrial and aquatic habitats [4]. Their dimorphic traits are exhibited in a myriad of ways, such as body size, body shape, skin texture, dermal ornamentation, vocal sacs, coloration, pheromone-producing glands, and chemosensory structures [5,6]. These dimorphic traits are permanent or seasonal. The permanent traits (e.g., body size, body shape, and vocal sacs) are mainly affected by developmental processes and growth before maturity [7], while the seasonal traits (e.g., dermal ornamentation, coloration, and pheromone-producing glands) are primarily influenced by hormones tightly associated with reproduction and the season [1]. In addition, some dimorphic traits, such as body size, morphological traits, dermal ornamentation, and coloration, are often quite easy to observe, but other traits may be more subtle, such as glandular structure, pheromone secretion, and epidermal texture [8]. Among these dimorphic traits, body size and body shape are the most common and the most extensively studied [9,10,11,12,13,14,15]. Sexual differences in body size are called sexual size dimorphism (SSD), while dimorphism in body shape is called sexual shape dimorphism (SShD). When males are larger than females, SD is called male-biased SD, whereas when females are larger than males, SD is called female-biased SD.

In a given environment, both sexes may be subject to similar selection pressures based on environmental characteristics, but at the same time they may be subject to very different sexual selection pressures that result in sexually dimorphic traits. Thus, SD is the consequence of different selection pressures acting differentially upon individuals of two sexes toward increased fitness [11,16]. Sexual selection, fecundity selection, and ecological selection are the most accepted mechanisms driving the evolution of SD. Sexual selection maximizes the reproductive fitness of the sexes [2], including intersexual (mate choice) and intrasexual (male–male competition) selection. Fecundity selection enlarges traits that improve reproductive ability [7,17]. Ecological selection results in divergent traits between sexes because of differences in the utilization of resources (habitat use, predation, and diet) [3,17] that maximize survival and growth [18]. Additionally, growing evidence suggests that SShD traits vary among populations within broadly distributed species [19,20] because of the different ecological conditions of each population. Therefore, SD studies among populations not only provide an understanding of the formation mechanism of SD and the degree of difference among populations, but also reflect the adaptations of SD traits to local environmental conditions.

The genus *Batrachuperus* (Urodela: Hynobiidae) is endemic to China and a total of six species have been recognized [21]. To date, SD was only studied in a population of *B. pinchonii* [14], and there is no report among multiple populations in species of this genus. The Chiala mountain salamander, *B. karlschmidti*, a hynobiid species endemic to China, lives in cold water and can be found under stones in small mountain streams from 1500 to 4250 m altitude in western Sichuan Province [21,22,23,24] and southeastern Gansu Province [25]. This species is poorly studied, as only skull morphometry [26], phylogeny [23,24,27], and hematology [28] have been studied. Due to the limited number specimens, research on skull morphology did not investigate SD [26]. Therefore, SD in *B. karlschmidti* has not been examined. In this study, we analyzed the SD in size and shape across three populations of *B. karlschmidti*. These three populations were chosen because they are represented by the greatest number of museum specimens in our collections, meeting the requirements for studying SD in terms of specimen quantity. The aims of this study were to (1) explore the SD in size and shape in each population, and explain SD based on existing theories; (2) explore the variations in SShD traits among populations, and analyze their causes. As the previous literature has indicated that *B. pinchonii*, a congeneric species of *B. karlschmidti*, has obvious SShD but does not have SSD [14], we hypothesized that (1) *B. karlschmidti* would exhibit SShD, but not SSD, and (2) that SShD traits would vary among populations.

## 2. Materials and Methods

### 2.1. Study Site

**Figure 1:**
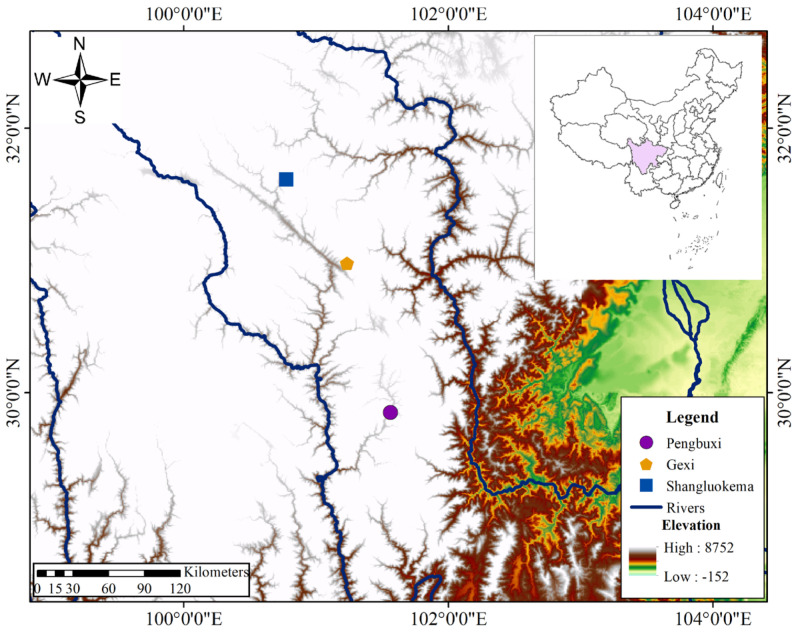
Localities of samples used in this study.

The *B. karlschmidti* specimens used in this study were collected from three populations situated in Sichuan Province within southwestern China: the Gexi, Shangluokema, and Pengbuxi populations (Figure 1). The environmental characteristics of these three populations are presented in Table 1.

### 2.2. Data Collection

**Figure 2:**
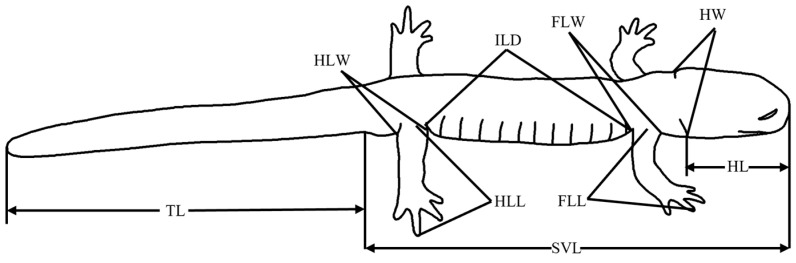
Characteristic dimensions of *Batrachuperus karlschmidti* used in this study. See the definitions and abbreviations for the characteristics in Table 2.

A total of 100 adult *B. karlschmidti* specimens (59 males and 41 females) were examined, preserved in 10% formalin at the Museum of Mianyang Normal University. These specimens were collected from the Pengbuxi population (11 males and 10 females) in June 2016, and the Gexi (16 males and 20 females) and Shangluokema populations (32 males and 11 females) in July 2017. As the breeding season of this species is from May to early August [29,30], the examined specimens represent individuals during the breeding period or post-breeding phase. Morphometric data for nine body characteristics (Table 2, Figure 2) were measured from each preserved specimen with a precision of 0.01 mm using digital calipers (Chixi, Chixi Corp., Shanghai, China); data were measured by the same person. These characteristics were chosen because they are commonly used in hynobiid salamander studies [10,14,31,32,33]. Sex was determined by examining the gonads through a small incision in the abdominal wall.

### 2.3. Statistical Analyses

To minimize deviations from normality and distortion effects caused by allometric relationships [34], all measurements were log10-transformed and then tested for normality (Kolmogorov–Smirnov test) and homogeneity of variance (Levene’s test). Since the log10-transformed variables were homogeneous (*p* > 0.05), the log10-transformed measurements were used for the following analyses. To analyze SSD, a two-way analysis of variance (ANOVA) was conducted with log10-transformed SVL as the dependent variable and sex and population as fixed factors to determine the effect of sex and population on SVL. To examine SShD, correlations between log 10-transformed shape variables and log10-transformed SVL were analyzed using the Spearman method. When all shape variables were highly correlated with SVL, the residuals, known as relative sizes, extracted from line regressions of each log 10-transformed shape variable against log-transformed SVL, were calculated to remove the effect of body size on each shape variable. Then, MANOVA was performed to test for the effect of sex and population on the residuals of the shape variables. The LSD test was used for post hoc comparisons. Results were considered significant if *p* ≤ 0.05. The measurements are presented as mean ± standard error. All statistical analyses were carried out in SPSS Statistics for Windows, version 22.0 (SSPS Inc., Chicago, IL, USA).

## 3. Results

The morphological measurements of *B. karlschmidti* are summarized in Table 3. Males had an SVL range of 59.41–95.11 mm and females of 60.24–93.89 mm. Males and females in each population had similar mean SVL values. Among the populations, the males and females of the Pengbuxi population had higher mean values, and the males and females of the Gexi and Shangluokema populations exhibited similar mean SVL measurements. Two-way ANOVA revealed significant differences in SVL among the populations (*F*_2,94_ = 32.562, *p* < 0.001), but no significant difference between sexes (*F*_1,94_ = 0.675, *p* = 0.413), and no significant interaction (*F*_2,94_ = 1.306, *p* = 0.276). The LSD tests indicated that SVL in males and females from the Pengbuxi population was significantly larger than in those from the Gexi (*p* < 0.001) and Shangluokema (*p* < 0.001) populations, while there was no significant difference between the latter two populations (*p* = 0.429).

Similarly to SVL, the mean shape variable values of males and females in each population are also roughly the same. The mean values of each shape variable in Pengbuxi population were all higher than those in Gexi and Shangluokema populations both in males and females, while the mean values of each shape variable in the Gexi and Shangluokema populations were similar in both males and females (Table 3).

**Figure 3:**
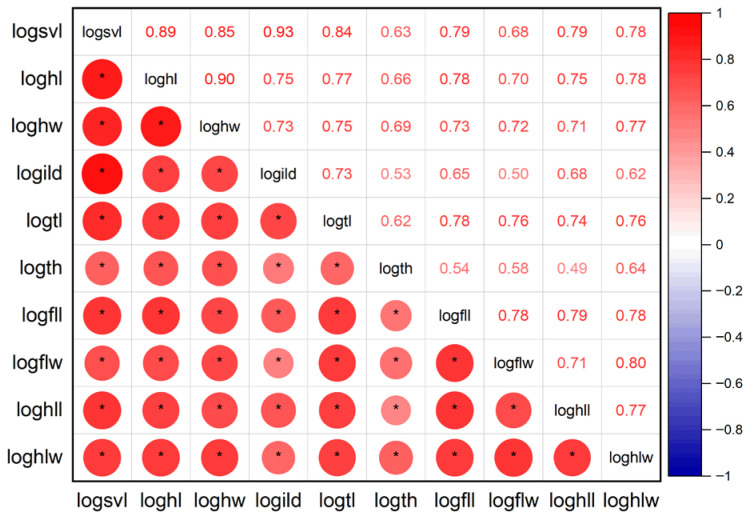
Pearson correlation coefficients between log10-transformed SVL and log 10-transformed shape variables. The descriptions of each abbreviation are listed in Table 2. The positive correlations are displayed in red and negative correlations in a blue color. The sizes of the circles are proportional to the correlation coefficients. * Represents significant correlation (*p* < 0.05).

All shape variables were highly correlated with SVL (*p* < 0.01 in all cases; Figure 3). The MANOVA results indicated significant differences in shape among populations (Wilks’ lambda = 0.441, *F*_18,172_ = 4.868, *p* < 0.001) and between sexes (Wilks’ lambda = 0.356, *F*_9,86_ = 17.269, *p* < 0.001), but no significant population × sex interaction (Wilks’ lambda = 0.667, *F*_18,172_ = 2.147, *p* = 0.006). As shown in Table 4, males were larger than females in relative HL, HW, TL, FLL, HLL, FLW, and HLW. In contrast, females were larger than males in relative ILD. The males in the Gexi population had larger relative HW than those in the Shangluokema population, but a smaller relative HLW than the Shangluokema and Pengbuxi populations. The Shangluokema population was smaller than the Gexi and Pengbuxi populations in relative HL, but larger in relative TL. The Pengbuxi population was smaller than the Gexi and Shangluokema populations in relative ILD (Table 5). On the other hand, relative HL and HW in females from the Pengbuxi population were larger than those from Gexi and Shangluokema, but smaller in relative TL. The relative HLL of the Gexi population was smaller than that of the Shangluokema and Pengbuxi populations, and the relative HLW of the Gexi population was smaller than that of the Shangluokema population (Table 5).

## 4. Discussion

*Batrachuperus karlschmidti* did not show obvious SD in body size, but had marked SD in head shape, trunk length, tail length, and limb shape. Additionally, the SShD traits differed among populations. These results support our hypothesis that *B. karlschmidti* exhibits obvious SShD, but not SSD, and the SShD traits varied among populations.

Body size is an important life history trait that influences almost all aspects of an individual’s biology [35], and SSD is a critical aspect in understanding the evolution of life history traits and mating systems [36]. Three types of SD pattern were reported in amphibian body size, including female-biased, male-biased, and no SSD [37]. Female-biased SSD is the most common pattern, which presents in 60.8% of the 79 urodele species and in 89.6% of the 589 anurans [37]. Male-biased SSD only presents in a few lineages, and some species show only weak or no SSD [36]. In general, male-biased SSD has been attributed to sexual selection, and female-biased SSD is due to fecundity selection, and no SSD is due to the combined effect of sexual selection and fecundity selection. None of the analyzed populations of *B. karlschmidti* exhibited obvious SSD. This SSD pattern has been reported in other salamanders, such as *Salamandra algira*, *Mertensiella caucasica* [38], and *B. pinchonii* [14]. This could have resulted from a balance between the effects of sexual and fecundity selection.

The head is the most important and complex part of an animal’s body, and head shape (HL and HW) is affected by lifestyle, feeding, and the environment. Both female-biased and male-biased SShD have been reported in the heads of salamanders. However, male-biased SShD is widely found, such as in *Eurycea aquatica* and *E. cirrigera* [5], *Pachyhynobius shangchengensis* [33], *Hynobius maoershanensis* [10], whereas female-biased SShD has only been reported in a few species, e.g., *B. pinchonii* [14]. SShD of the head may be due to selection, fecundity, or ecological selection. Sexual selection favors males with larger heads to enhance competitive ability between males and increase reproductive success [39]. Fecundity and ecological selection favor females with large heads to consume more energy for reproductive investment [3,14]. Here, male-biased SShD in the head of *B. karlschmidti* was detected in the Gexi population, which may have been due to sexual selection.

Trunk length (which here refers to interlimb distance) represents abdominal volume [14]. Fecundity selection is responsible for female-biased SShD in trunk length, which is a common phenomenon and widely reported in salamanders, such as *Onychodactylus zhangyapingi* [32], *P. shangchengensis* [33], and *B. pinchonii* [14]. A long trunk length accommodates a larger abdominal volume to produce/store larger ovaries and more ova [12,17], thereby improving the fecundity of females [39]. All analyzed populations of *B. karlschmidti* showed marked female-biased SShD in trunk length, which may be attributed to fecundity selection.

Limbs are the main locomotor organs of salamanders, and they also include the function of reproduction. For example, males of most species of internally fertilized salamanders use their limbs to grip the females during amplexus, and to prevent a takeover by a competing male [6], whereas males of most species that externally fertilize use their limbs to grasp females, hold and embrace the egg sacs, and resist fertilization interference by other males [33,40,41]. These functional demands of males result in male-biased SShD of the limbs. *Batrachuperus karlschmidti* is a species of externally fertilized salamander in which the limbs are used to grasp females and hold and embrace the egg sacs during reproduction. Longer and stronger limbs increase reproductive and competitive abilities. Thus, male-biased SShD of the limbs in *B. karlschmidti* may be explained by sexual and fecundity selection.

The tail is an important organ for salamanders, as it has several functions, such as locomotion, energy storage, defense, respiration, and reproduction [6,42,43]. A stronger (longer, wider, and higher) tail generally means better mobility, defense, breathing, energy storage, and reproductive abilities. SShD of the tail is explained by the demands of energy storage and reproductive success. Males/females have shorter or narrower tails when they invest more energy in reproduction than storage [42]. In many salamanders, the male uses his tail during courtship, wafting glandular secretions toward the female or stroking the female [43]. Longer tails improve the ability of male reproduction [10,42]. In this study, two populations of *B. karlschmidti* showed noticeable male-biased SShD of the tail. Though the reproductive biology of *B. karlschmidti* has not been reported, males with longer tails in these two populations are explained by fecundity selection.

A previous study reported that SShD traits vary among populations of widespread species, e.g., *Triturus marmoratus* [15]. This phenomenon was demonstrated in this study. For example, SShD in HL, HW, and HLL was only present in the Gexi population, and SShD of TL was absent in the Gexi population (Table 3). These differences may be explained by the dissimilar levels of selection forces in the environments where the populations occur, such as the sex ratio, as well as food and microhabitat abundance. For instance, in populations with a higher proportion of males, intensified male–male competition leads to selective pressures favoring traits associated with competition and reproductive success. The differences in SShD traits among populations may reflect their adaptations to the local environment. In addition, age structure and growth rate were also regarded as potential factors contributing to SD [44]. For example, head size dimorphism in *Aneides flavipunctatus* was attributed to a higher head growth rate in males at sexual maturity relative to females [45]. However, the population dynamics, ecology, reproductive behavior, and life history of this species have not been reported; thus, future studies should be carried out to test whether these factors explain the differences in SShD traits among populations. For example, whether the sex ratio and food abundance lead to male-biased head and limb dimorphism, whether the differences in SShD traits are relate to the age and growth rate.

## 5. Conclusions

This study examined the SD in size and shape among three populations of *B. karlschmidti*. SD was observed in body shape but not in body size. Head shape, limb shape, and tail length showed obvious male-biased SShD, and interlimb distance revealed a marked female-biased SShD. Differences in SShD traits were found among populations. This study demonstrated that SD of different morphological traits is a consequence of different selective forces acting differently on the sexes. Although existing theories try to explain SShD in this species, future studies about the reproductive behavior, population dynamics, ecology, and life history of *B. karlschmidti* should be carried out to confirm the explanations.

## Figures and Tables

**Table 1:** Environmental characteristics from three populations of *Batrachuperus karlschmidti*.

Characteristics	Populations		
	Gexi	Shangluokema	Pengbuxi
Coordinates	101°13′22.36″ E, 30°58′10.55″ N	100°46′40.85″ E, 31°37′0.30″ N	101°33′44.12″ E, 29°47′26.11″ N
Elevation (m)	3622	3644	3743
Average annual precipitation (mm)	667	669	737
Average annual temperature (°C)	2.46	2.54	3.93

**Table 2:** Definitions of the morphological characteristic set and abbreviations used to characterize *Batrachuperus karlschmidti*.

Characteristics	Abbreviations	Definition
snout-vent length	SVL	from the tip of snout to the posterior margin of the cloaca
head length	HL	from the tip of the snout to the gular fold
head width	HW	width of the head at its widest point
tail length	TL	from the posterior margin of the cloaca to the tip of the tail
forelimb length	FLL	from the base of the forelimb to the tip of the longest finger
hindlimb length	HLL	from the base of the hindlimb to the tip of the longest toe
Interlimb distance	ILD	distance between the posterior base of the forelimb (axilla) to the anterior base of the hindlimb (groin) on the same side
forelimb width	FLW	maximum width of the forelimb
hindlimb width	HLW	maximum width of the hindlimb

**Table 3:** Descriptive statistics (mean ± S.E.) of original morphometric characteristics (mm) of males and females *Batrachuperus karlschmidti* from three populations.

Characteristics	Gexi		Shangluokema		Pengbuxi	
	Males (*n* = 16)	Females (*n* = 20)	Males (*n* = 32)	Females (*n* = 11)	Males (*n* = 11)	Females (*n* = 10)
SVL	68.88 ± 1.05 (62.03–76.66)	72.29 ± 1.38 (64.20–85.64)	72.03 ± 0.98 (59.41–82.37)	71.01 ± 1.62 (60.24–77.98)	82.84 ± 2.36 (66.01–95.11)	83.43 ± 1.76 (73.18–93.89)
HL	18.85 ± 0.37 (16.32–21.35)	18.57 ± 0.23 (17.13–20.91)	18.68 ± 0.23 (15.34–21.28)	18.19 ± 0.35 (16.58–20.13)	21.69 ± 0.50 (18.37–24.33)	21.55 ± 0.39 (19.92–23.55)
HW	13.51 ± 0.32 (11.06–15.70)	13.15 ± 0.28 (11.26–16.17)	13.41 ± 0.20 (10.51–16.21)	12.91 ± 0.21 (12.07–14.03)	15.32 ± 0.38 (13.01–17.77)	15.67 ± 0.36 (13.59–17.41)
TL	56.10 ± 1.66 (45.25–67.68)	58.35 ± 1.38 (50.16–77.01)	63.88 ± 1.44 (45.40–77.55)	58.89 ± 2.49 (45.76–70.68)	70.11 ± 2.10 (56.61–83.05)	64.53 ± 1.68 (55.18–72.59)
FLL	17.37 ± 0.32 (15.01–20.18)	16.85 ± 0.28 (14.62–19.36)	18.04 ± 0.23 (14.94–20.81)	16.67 ± 0.44 (14.50–18.67)	20.10 ± 0.36 (17.87–21.84)	18.76 ± 0.34 (17.32–20.91)
HLL	20.38 ± 0.30 (18.63–23.03)	20.09 ± 0.40 (16.90–23.82)	21.60 ± 0.33 (18.22–25.34)	20.86 ± 0.49 (17.73–23.63)	23.93 ± 0.48 (20.00–25.89)	23.63 ± 0.50 (21.24–25.27)
ILD	30.19 ± 0.44 (27.38–33.08)	33.51 ± 0.83 (27.69–41.10)	32.43 ± 0.55 (26.30–39.23)	32.94 ± 0.92 (28.56–37.79)	35.90 ± 1.21 (28.16–42.30)	38.83 ± 1.19 (31.10–43.42)
FLW	3.02 ± 0.11 (2.33–4.15)	2.57 ± 0.10 (1.90–3.34)	3.26 ± 0.07 (2.09–3.99)	2.62 ± 0.10 (1.88–3.08)	3.81 ± 0.16 (2.37–4.28)	3.20 ± 0.12 (2.71–4.03)
HLW	4.09 ± 0.14 (3.35–5.30)	3.95 ± 0.14 (3.10–5.38)	4.58 ± 0.09 (3.36–5.72)	4.13 ± 0.16 (3.26–4.99)	5.70 ± 0.24 (4.51–6.70)	5.05 ± 0.11 (4.52–5.66)

**Table 4:** Means of residuals from regressions of log10-transformed body shape characteristics vs. log10-transformed snout-vent length (SVL) of males and females *Batrachuperus karlschmidti* from the analyzed populations, and the results of MANOVA models. Males larger than females (pattern M); females larger than males (pattern F).

Characteristics	Gexi				Shangluokema				Pengbuxi			
	Males	Females	*p*	Pattern	Males	Females	*p*	Pattern	Males	Females	*p*	Pattern
HL	0.796	−0.437	0.001	M	−0.250	−0.604	0.144		0.652	0.346	0.489	
HW	0.756	−0.486	<0.001	M	−0.062	−0.483	0.129		0.108	0.372	0.600	
TL	−0.268	−0.422	0.554		0.810	−0.091	0.004	M	0.015	−1.236	<0.001	M
FLL	0.389	−0.758	0.001	M	0.504	−0.751	<0.001	M	0.700	−0.663	0.001	M
HLL	0.025	−0.842	0.020	M	0.390	0.013	0.170		0.337	0.011	0.409	
ILD	−0.540	0.589	<0.001	F	−0.051	0.667	0.019	F	−1.175	0.407	0.002	F
FLW	0.567	−1.069	<0.001	M	0.700	−0.719	<0.001	M	0.512	−0.778	<0.001	M
HLW	−0.007	−0.938	0.003	M	0.521	−0.282	0.001	M	0.808	−0.360	0.017	M

**Table 5:** Comparison results of means of residuals from regressions of each log-transformed shape characteristics vs. log10-transformed snout-vent length (SVL) within males and females *Batrachuperus karlschmidti* from the analyzed populations, and the results of MANOVA models with LSD post hoc test. Significant differences in bold. Gexi (G), Shangluokema (S), and Pengbuxi (P).

Characteristics	Males			Females		
	G vs. S	G vs. P	S vs. P	G vs. S	G vs. P	S vs. P
HL	**0.000**	0.667	**0.004**	0.626	**0.032**	**0.022**
HW	**0.005**	0.075	0.597	0.994	**0.023**	**0.043**
TL	**0.000**	0.328	**0.003**	0.301	**0.017**	**0.004**
FLL	0.602	0.273	0.438	0.984	0.789	0.826
HLL	0.174	0.362	0.862	**0.024**	**0.029**	0.997
ILD	0.050	**0.047**	**0.000**	0.812	0.594	0.499
FLW	0.471	0.813	0.371	0.209	0.310	0.852
HLW	**0.047**	**0.017**	0.338	**0.027**	0.057	0.817

## Data Availability

Data are contained within the article.
